# A chromosome-scale assembly of the smallest Dothideomycete genome reveals a unique genome compaction mechanism in filamentous fungi

**DOI:** 10.1186/s12864-020-6732-8

**Published:** 2020-04-23

**Authors:** Bo Wang, Xiaofei Liang, Mark L. Gleason, Tom Hsiang, Rong Zhang, Guangyu Sun

**Affiliations:** 1 State Key Laboratory of Crop Stress Biology in Arid Areas and College of Plant Protection, Northwest A&F University, Yangling, 712100 Shaanxi Province China; 2 MOE Key Laboratory for Intelligent Networks & Network Security, Faculty of Electronic and Information Engineering, Xi’an Jiaotong University, Xi’an, 710049 China; 3 Department of Plant Pathology and Microbiology, Iowa State University, Ames, IA 50011 USA; 4 School of Environmental Sciences, University of Guelph, Guelph, Ontario N1G 2W1 Canada

**Keywords:** Compact genome, Genome architecture, Ectophytic, Extreme environment fungi, Oxford Nanopore sequencing, Retroelement

## Abstract

**Background:**

The wide variation in the size of fungal genomes is well known, but the reasons for this size variation are less certain. Here, we present a chromosome-scale assembly of ectophytic *Peltaster fructicola*, a surface-dwelling extremophile, based on long-read DNA sequencing technology, to assess possible mechanisms associated with genome compaction.

**Results:**

At 18.99 million bases (Mb), *P. fructicola* possesses one of the smallest known genomes sequence among filamentous fungi. The genome is highly compact relative to other fungi, with substantial reductions in repeat content, ribosomal DNA copies, tRNA gene quantity, and intron sizes, as well as intergenic lengths and the size of gene families. Transposons take up just 0.05% of the entire genome, and no full-length transposon was found. We concluded that reduced genome sizes in filamentous fungi such as *P. fructicola*, *Taphrina deformans* and *Pneumocystis jirovecii* occurred through reduction in ribosomal DNA copy number and reduced intron sizes. These dual mechanisms contrast with genome reduction in the yeast fungus *Saccharomyces cerevisiae*, whose small and compact genome is associated solely with intron loss.

**Conclusions:**

Our results reveal a unique genomic compaction architecture of filamentous fungi inhabiting plant surfaces, and broaden the understanding of the mechanisms associated with compaction of fungal genomes.

## Background

By the early twenty-first century, sequencing of the human genome was complete [1]. The total number of human genes was predicted to be nearly 25,000 [2]. Because the DNA which encoded proteins accounted for only 1.0% ~ 1.5% of the total DNA, the human genome was characterized as a *C*-value paradox; that is, not compact [3]. In contrast, the genome of the pufferfish (*Fugu rubripes*) is one-eighth the size of the human genome but it has a similar gene repertoire, so it was classified as a compact-genome vertebrate [4, 5]. In fungi, the yeast *Saccharomyces cerevisiae* possesses a highly compact genome because of significant intron loss compared to filamentous fungi [6]. The filamentous fungi *Pneumocystis* spp. and *Taphrina deformans*, both of the Taphrinomycotina subphylum, were also recognized to have compact genome structures [7–9]. The *Pneumocystis* genome exhibits substantial reduction of intron size, ribosomal RNA gene copy number and metabolic pathways [9], whereas *T. deformans* contains few repeated elements and short intron size, specially, just one ribosomal RNA gene copy [8].

The habitats of compact-genome species are usually extreme environments. It is therefore reasonable to hypothesize that streamlining of genome size and function is driven by restrictions imposed by their lifestyles [3]. Fungi in the sooty blotch and flyspeck (SBFS) complex exclusively colonize plant surfaces, which are extreme environments characterized by prolonged desiccation, nutrient limitation, and exposure to solar radiation [10]. Recent research has presented compelling evidence that SBFS fungi underwent profound reductive evolution during the transition from plant-penetrating parasites to plant-surface colonists [11–14].

Fungal genomes are usually smaller than most animal and plant genomes. It was found that fungal genomes were very diverse in nature varies from 8.97 Mb to 177.57 Mb [15]. The average genome sizes of Ascomycota and Basidiomycota fungi are 36.91 and 46.48 Mb respectively [15]. The class Dothideomycetes, one of the largest groups of fungi with a high level of ecological diversity, had the average genome sizes of 38.92 Mb, ranged from the smallest 21.88 Mb in *Baudoinia compniacensis* to the largest 177.57 Mb in *Cenococcum geophilum* [15, 16].

Our recently published draft nuclear genome of a representative SBFS fungus, *Peltaster fructicola* was 18.14 Mb, which has the smallest fungal genome known among Dothideomycetes. The genomic analysis of *P. fructicola* revealed several unique features, including a very small repertoire of repetitive elements and very few plant-penetrating genes, such as those involved in plant cell wall degradation, secondary metabolism, secreted peptidases, and effectors, and showed that the gene number reduction made this genome among the smallest in filamentous fungi [12]. In this study, we aim to achieve whole chromosome sequence assemblies for *P. fructicola* genome using Oxford Nanopore long read sequencing technology and to uncover the possible genome compaction mechanism by comparing to other filamentous fungal genomes.

## Results

### Chromosome-scale genome sequence assembly

**Fig. 1:**
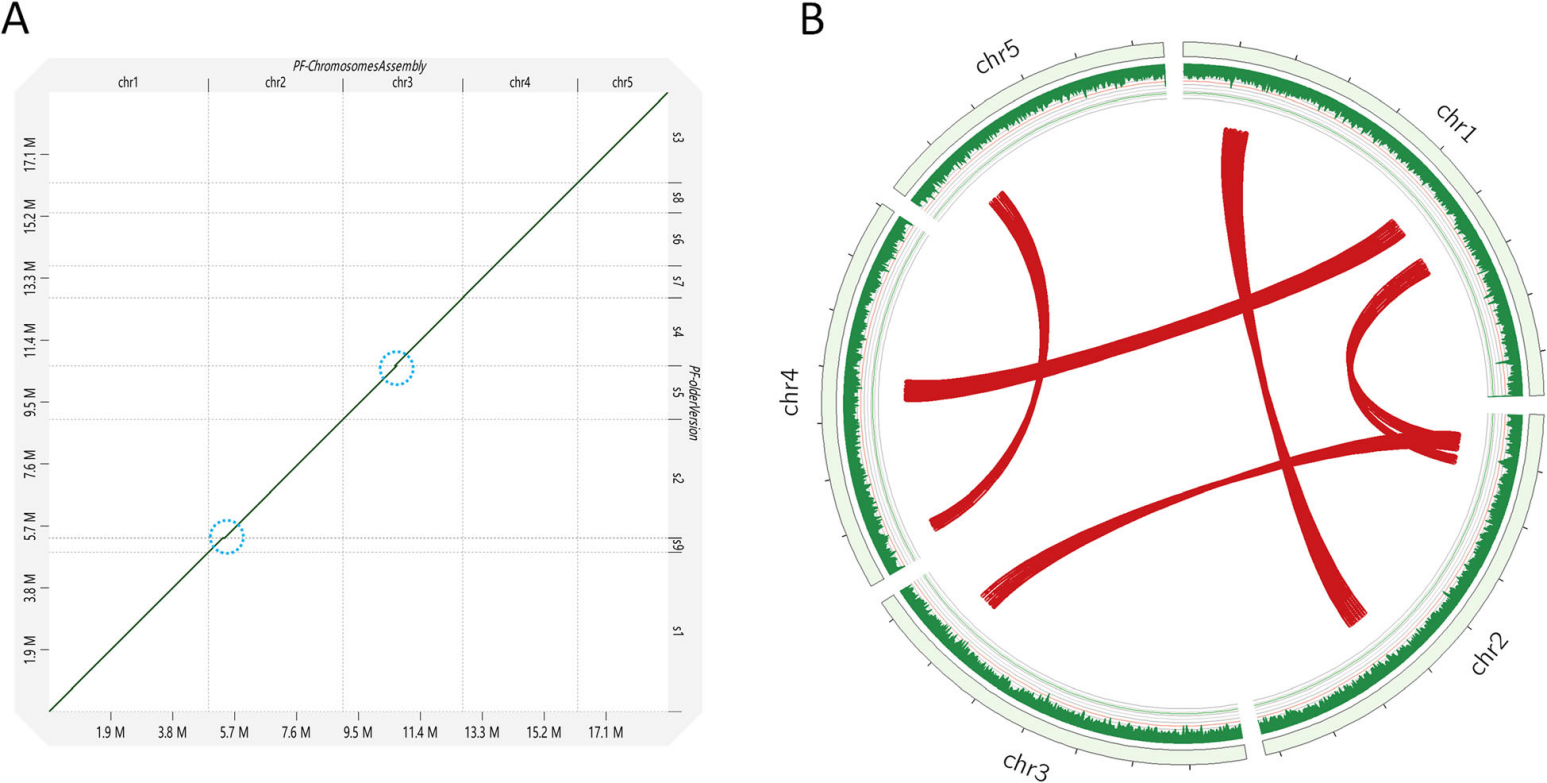
Chromosome level assembly of *P. fructicola* genome and syntenic blocks of the five chromosomes. **a**. Dot plot illustrating the comparative analysis of the chromosome level assembly genome and previous draft genome [12]. Scaffolds were grouped into chromosomes. The blue circles highlight major linker regions in chromosome level genome version. **b**. Circos plot displaying five collinearity blocks among five chromosomes of *P. fructicola*. From outside to inside, it represents the distribution of chromosome display, GC contents and syntenic regions, respectively

**Fig. 2:**
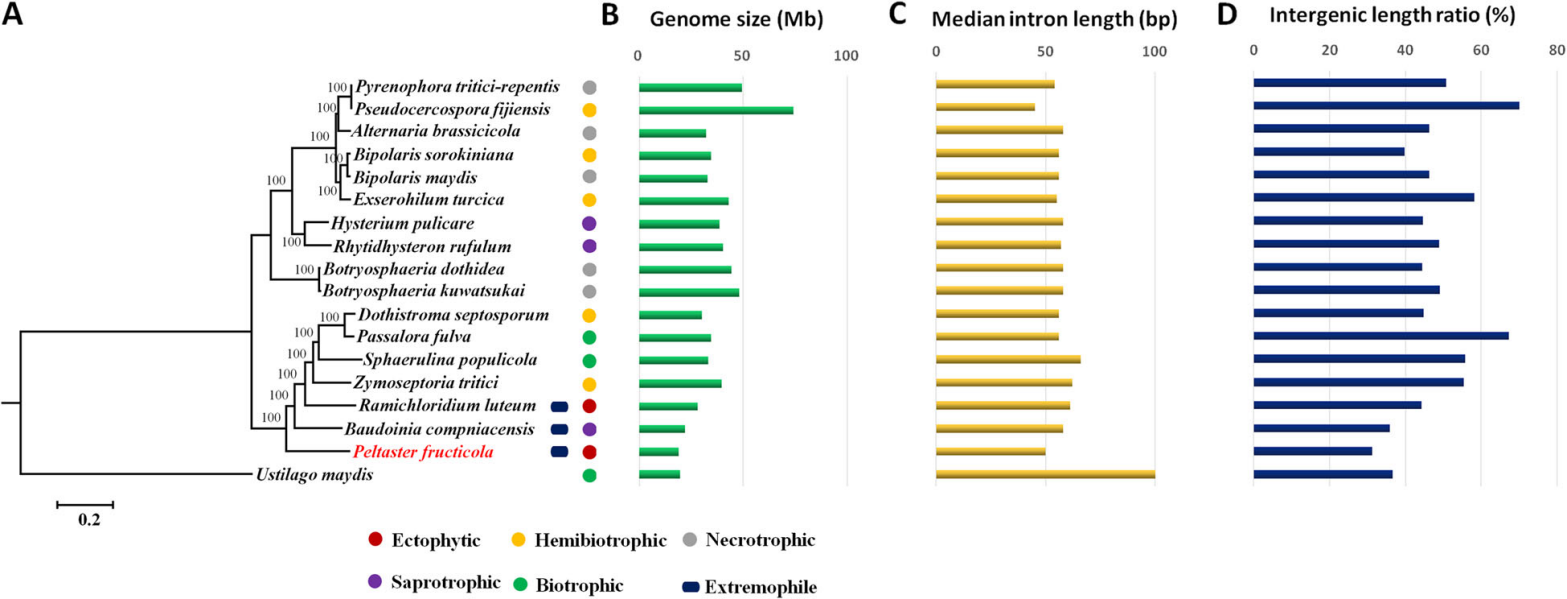
Phylogeny and genome characteristics of *Peltaster fructicola* and other 16 studied Dothideomycetes species. **a.** A maximum likelihood phylogenetic tree constructed from concatenated alignment of 1957 single-copy orthologs conserved across all species. Bootstrap values are indicated on branches. *Ustilago maydis* with small genome was used as the outgroup. **b.** Genome size compared among selected species. **c.** Median length of introns compared among selected species. **d.** Intergenic length ratio (%) compared among selected species

Oxford Nanopore single-molecule sequencing using one flow cell produced 7.71 Gb of raw sequence data, and average length of passed reads was 19,278 bp. After quality and length filtering, the remaining reads provided approximately 406 fold genome coverage. The 369,827 error-corrected reads (N50 length = 26,789 bp) were assembled using our “assemble and polish pipeline” to give an assembly of 6 unitigs. Five of the six unitigs were completely sequenced from telomere to telomere without gaps (Fig. 1). The additional unitig was the circular mitochondrial genome. The size of the final assembled nuclear-genome was 18.99 Mb, with a N50 length of 3.68 Mb, which was composed of five chromosomes ranging from 2.77 Mb to 4.89 Mb. The five telomere-to-telomere chromosomes were categorized as pf_chr1 to pf_chr5, from the largest to the smallest. The genome size was close to the assembly size only using Illumina short-read sequencing (18.14 Mb) and theoretical size (19.54 Mb) [12]. The genome of *Peltaster fructicola* is smaller than the extremophilic sooty mold *Baudoinia compniacensis* (21.88 Mb) (Fig. 2a-b), which was the smallest previously reported genome for a filamentous fungus in Dothideomycetes [16].

**Fig. 3:**
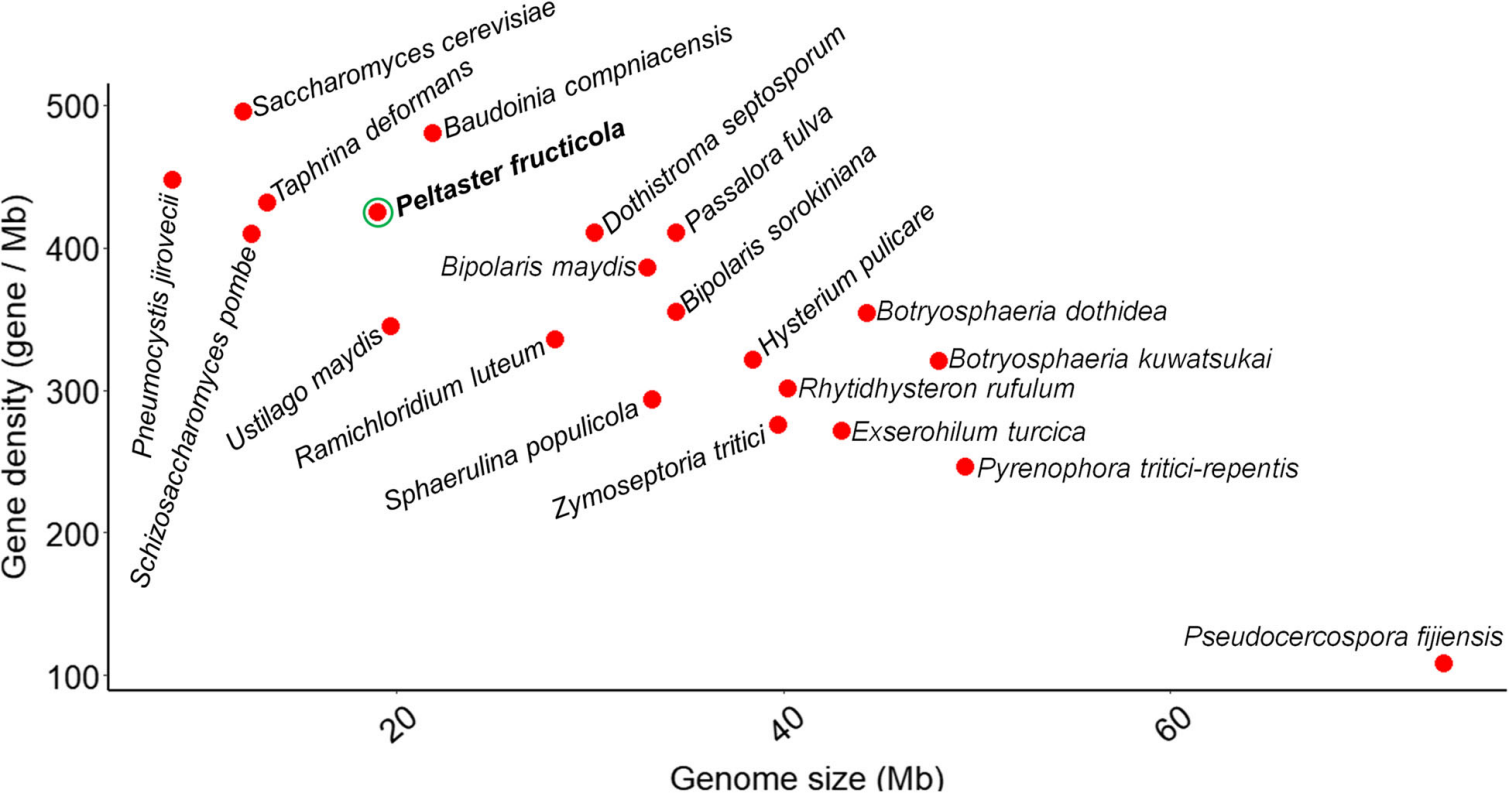
Comparison of gene density and genome sizes in selected species

A relatively small number of protein-coding genes was annotated in *P. fructicola* (8072) (average size = 500 aa) (Fig. S1), compared with the fungal phytopathogens *Sphaerulina populicola* (9739) and *Passalora fulva* (14,127). *P. fructicola* has higher gene density than other characterized Dothideomycetes species, except for *B. compniacensis* (Fig. 3). The genomic size of *P. fructicola* is similar to that of the basidiomycete *Ustilago maydis* (19.66 Mb) [17], but *P. fructicola* has higher gene density (425 per Mb vs. 345 per Mb) and shorter average intron (Fig. 2c and Fig. S2) and intergenic length (Fig. 2d). There is little difference in gene density between *P. fructicola* and compact fungal genome of *Pneumocystis jirovecii* [9] (425 per Mb vs. 448 per Mb), or with fungus *Taphrina deformans* (431 per Mb), but exceeded most of fungi examined (Fig. 3).

Of the 8072 gene models for *P. fructicola*, 8057 were supported by at least one FPKM (Fragments per kilobase of exon per million reads mapped), and 7658 models were supported by at least 10 FPKM. Among the predicted genes, 6010 genes had matches to entries in the PFAM database, 7575 genes had matches in the non-redundant database and 5723 were mapped to Gene Ontology (GO) terms (Fig. S3). We re-predicted a previous draft genome of *P. fructicola* [12] using the pipeline developed in this study (see methods section) and obtained 7604 gene models. To compare gene content between the current and former annotations of *P. fructicola*, we used BUSCO v.1.2 to search for a set of 1438 fungi universal single-copy orthologous genes (FUSCOGs). Among 1438 FUSCOGs, the proportion classified as ‘fragmented’ declined from 5.8% in the previous annotation to 3.8% in the current annotation, and the proportion classified as ‘missing’ declined from 1.8 to 1.1%. Some fragmented and missing regions were recovered in this new assembly version (Fig. 1a). The BUSCO identification of nearly all (99%) core fungal genes of the current annotation of *P. fructicola* suggested a high-quality assembled genome and predicted gene set.

### Telomere repeat

Chromosome-scale assembly suggested that the repeat unit in *P. fructicola* telomeres was TAGGG. This unit was has not been previously reported from other fungi (Telomerase Database: http://telomerase.asu.edu/sequences_telomere.html), but was reported from the unicellular heterotrophic flagellate *Giardia intestinalis* [18], a unicellular heterotrophic flagellate, whose genome is compact [19]. A repeat unit of telomeres of *P. fructicola* and *Giardia* spp., formed by five bases, is the shortest compared to all other eukaryote species reported (6–26 bases) (Telomerase Database: http://telomerase.asu.edu/sequences_telomere.html)*.* Interestingly, none of the subtelomeric regions (up to 25 kb) in the *P. fructicola* nuclear genome showed homology to each other (*e*-value = 1e-3, coverage > 10%). This situation is different from that of *Saccharomyces cerevisiae*, in which all chromosomal ends contain core X elements [20]. In addition, all subtelomeric regions in *P. fructicola* were of low gene density with only 30 genes detected in the 10 subtelomeric regions composed of 250 kb (Table S1) resulting in 0.12 genes per kb. In contrast, the average whole genome gene density was 0.425 genes per kb. There was no regularity in the distribution of genes in the subtelomeric region on chromosomes, and most of the genes had unknown functions. The pf_chr2 right arm contained a GH31 gene and pf_chr5 right arm contained an amino acid permease (Table S1). The function of the GH31 gene was predicted to be alpha-glucosidase activity, which can release glucose from the non-reductive end of oligosaccharide substrates by cutting alpha-1,4-glycoside bonds [21]. Amino acid permease is a membrane protein with 12 transmembrane domains whose function is to transport amino acids into cells. Using Phobius software (http://phobius.binf.ku.dk/), we predicted that the g6510.t1 gene had 12 transmembrane domains, which further confirmed that the gene was an amino acid permease.

### Decreased chromosome number and relative independence of five chromosomes

**Fig. 4:**
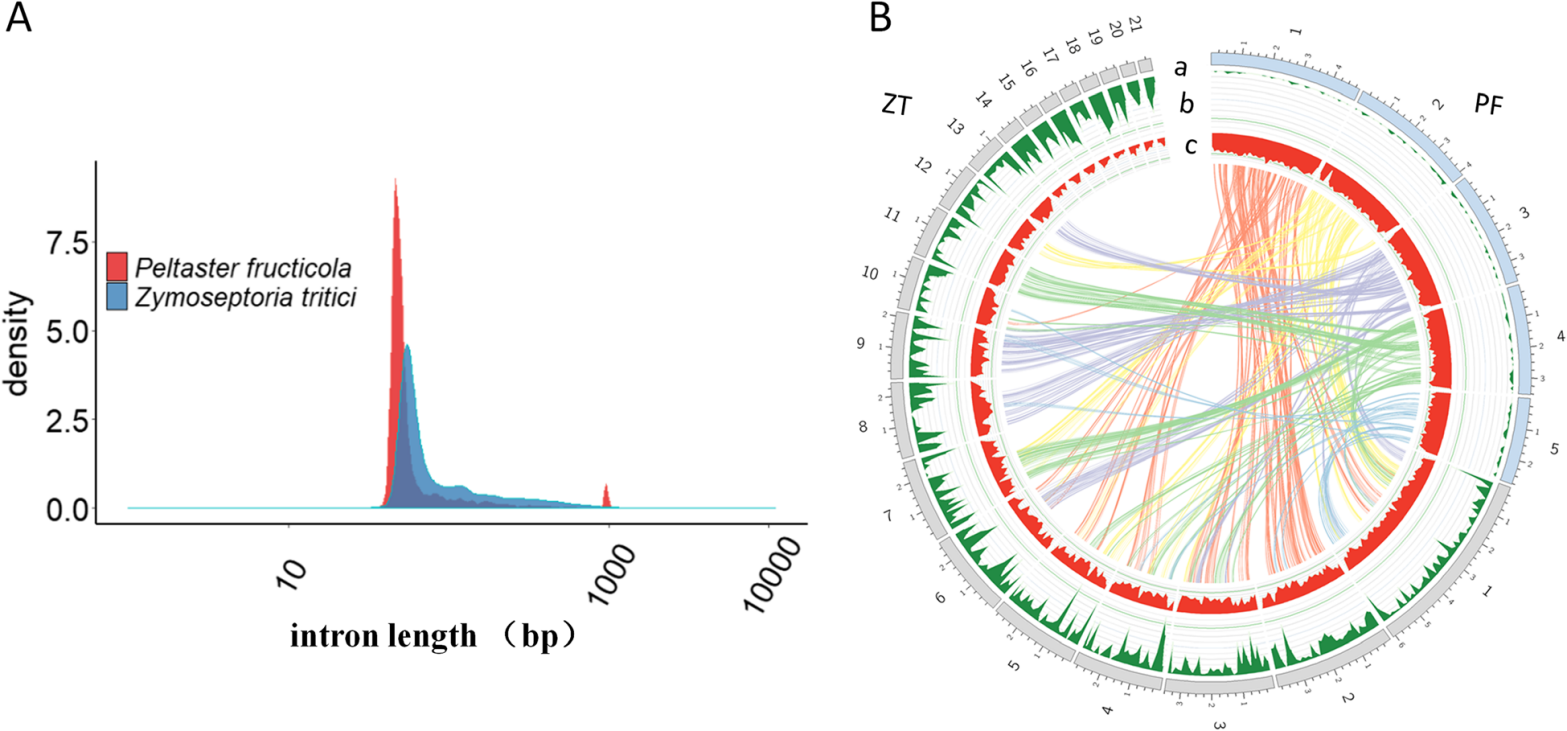
Length of intergenic region, colinearity, transposable elements (TEs) and gene density analysis between *Peltaster fructicola* and *Zymoseptoria tritici*. **a**. Intergenic length density plot of *P. fructicola* genome and *Z. tritici* genome. **b**. Syntenic blocks between two species are shown in various color lines (BLASTN coverage > 1 kb). *P. fructicola* (PF) chromosomes are shown as light blue colour, *Z. tritici* (ZT) 21 chromosomes [22] are shown as colour. Track a-c are the distribution of chromosomes, TEs density and gene density respectively, with densities calculated in 100 kb windows

The finished genome of *P. fructicola* contained five chromosomes, much fewer than the 21 chromosomes of its closely related plant-penetrating species, *Zymoseptoria tritici* [22]*.* We found that the *P. fructicola* genome was overall gene-dense with shorter intron (Fig. 2c) and intergenic lengths (Fig. 4a) but longer exon lengths compared to *Z. tritici* genome (exon size median: 328 vs. 300) which shows overall gene-sparse (Fig. 4b). Pairwise sequence comparison of the genomes of *P. fructicola* with *Z. tritici* (Fig. 4b) revealed a high degree of micro-mesosynteny (genome segments having a similar gene content but shuffled order and orientation), likely due to intrachromosomal rearrangements [23]; this level of rearrangement appears to be among the most striking between closely related genera anywhere in the Dothideomycetes [24]. There were no syntenic regions observed between the *P. fructicola* chromosomes and the eight accessory chromosomes of *Z. tritici* (Fig. 4b)*.* Chromosomal fusion may have led to depletion in numbers of *P. fructicola* chromosomes. For example, fusional DNA may have carried a gene that was beneficial to the recipient species, and thus the chromosome (or a large section) carrying this gene may have been retained while sections not essential for environmental adaptation were lost; these processes may help to explain both *P. fructicola*’s massive loss of pathogenicity-related genes and its retention of cutinase and secreted lipases [12, 24].

The pf_chr5 had greater density than the other four chromosomes, whereas rDNA repeat units gave pf_chr2 the lowest gene density. Only 69 collinear genes (0.85% of all genes) were detected, in five collinearity blocks (Fig. 1b). One pair of collinear genes located on pf_chr1 and pf_chr2 were involved in DNA repair (Table S2).

### Very low repeat content

**Fig. 5:**
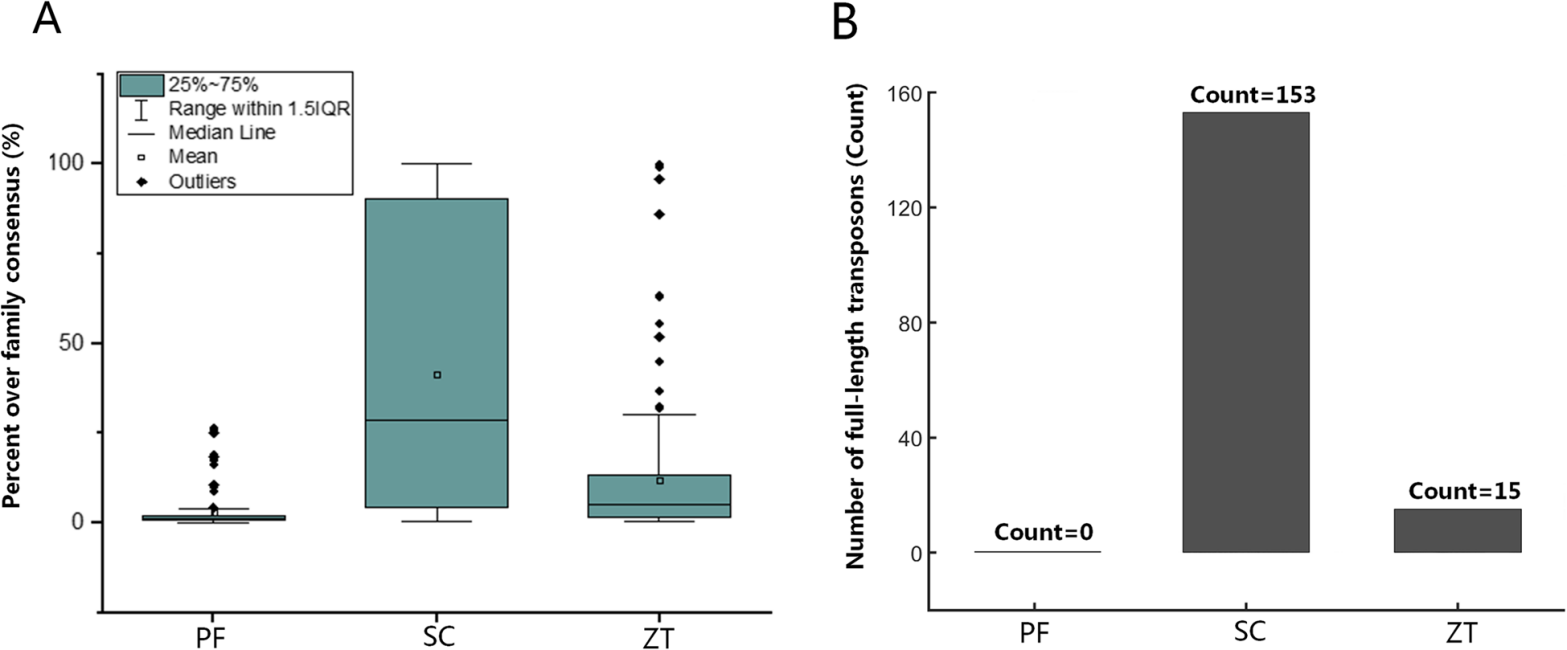
Transposons length analysis of *P. fructicola* (PF) compared with *S. cerevisiae* (SC) and *Z. tritici* (ZT). **a**. Boxplots of proportion of total TE length*.* **b.** Number of full-length transposons are shown (> 90% length over family consensus)

Multivariate repeated DNA sequences may account for variations in genome size [25]. Analysis of the repeat content of the chromosome-scale assembly of *P. fructicola* revealed that repeat elements comprised only 0.34%. When compared to other highly compact fungi, the repeat content of *P. fructicola* was also the lowest (Table 2). Most of the repeat elements identified were found in simple repeat sequences (0.278%) (Table S3). Only 0.05% of the genome assembly was classified as transposable element (TE) insertions. A total of 112 TE insertion locations were of multiple origins, representing 11 TE families from the two main TE orders (Class I/retrotransposons and Class II/DNA transposons). Most of the TE insertions were from retroelements (86.6%), which were created based on the three primary ingredients: *Ty1*/*Copia* long terminal repeats (LTR) elements, *Gypsy*/*DIRS1* LTR elements and *Tad1* long interspersed nuclear elements (Table 1). The *Gypsy* and *Copia* superfamilies were the main LTR-retrotransposon elements (Table 1). Maximum length percentage of total TE length were only 27% (According to RepBaseEdition-20,170,127) (Fig. 5a), so no full-length TE was detected in the *P. fructicola* genome (Fig. 5b), and the lengths were very short (Table S3). A total of nine Class II TE families [i.e., 3 *hobo-Activator*, 1 *Helitron,* 1 *TcMar-Sagan*, 1 *TcMar-Pogo*, 2 *TcMar-Fot1*, 1 *En-Spm*, 4 *Harbinger*, 1 *P-element* and one unclassified element] were identified. The fragment length of DNA transposons was only 17% of full length extracted from RepBaseEdition-20,170,127 (https://www.girinst.org/). The pf_chr4 had the most TE elements (*n* = 31) compared to pf_chr1 (*n* = 22), pf_chr2 (*n* = 21), pf_chr3 (*n* = 24), and pf_chr5 (*n* = 15). The number of DNA transposons was similar to that of *S. cerevisiae* but the number of retroelements was significantly lower (Table 1)*.* When compared to TE families in *Z. tritici*, we found that *P. fructicola* had a reduced battery of Class I and Class II transposable elements (Table 1).

**Table 1:** Classified repeat contents in *Peltaster fructicola*, *Saccharomyces cerevisiae* and *Zymoseptoria tritici*. All annotation data were analysis using the pipeline described in method section

	*P. fructicola*	*S. cerevisiae*	*Z. tritici*
**Genome assembly source**	Current study	NCBI R64	NCBI IPO323
**Retroelements**	**97**	**562**	**2261**
**LINEs (long interspersed nuclear elements):**	5	4	537
Penelope	0	0	1
Tad1	5	3	376
RTE/Bov-B	0	0	97
L1/CIN4	0	0	63
CRE	0	1	0
**LTR (long terminal repeats) elements:**	92	558	1376
BEL/Pao	0	0	37
Ty1/Copia	34	477	347
Gypsy/DIRS1	57	81	991
Ngaro	0	0	1
Retroposon (Unclassified)	1	0	348
**DNA transposons**	**15**	**18**	**564**
hobo-Activator	3	0	85
hAT-hATw	1	0	0
hAT-Ac	2	0	1
hAT-Restless	0	0	84
Helitron	1	1	1
TcMar-Sagan	1	0	1
TcMar-Pogo	1	0	0
TcMar-Fot1	2	1	88
TcMar-Tc1	0	0	40
TcMar-Ant1	0	1	63
Novosib	0	0	30
Dada	0	0	1
En-Spm	1	7	2
DNA (Unclassified)	1	4	44
MuLE-MuDR	0	0	63
Tourist/Harbinger	4	3	61
Other (Mirage, P-element, Transib)	1	1	0
Simple repeats:	1279	2828	6392
Low complexity:	78	530	830
**Transposon content (%)**	**0.05**	**3.41**	**10.42**
**Repeat content (%)**	**0.34**	**5.13**	**12.26**

### Reduced rDNA and tRNA genes

Because of the strong positive relationship between rDNA copy number and genome size [26], we examined rDNA copy number to determine its relationship to the small genome size of *P. fructicola*. The *P. fructicola* rDNA unit was defined according to the complete rDNA sequence of *Neurospora crassa* (GenBank accession: FJ360521) using BLASTN. We obtained a 5932 bp rDNA unit including 18S–5.8S-28S ribosomal genes, which were located on pf_chr2. Like most eukaryotic species [27], 5S rDNA genes of *P. fructicola* were found outside the rDNA units, and were situated on pf_chr1, 3, 4 and 5. We estimated nine copies of the rDNA gene cassette in *P. fructicola* according to a computational method using whole-genome short-read DNA sequencing [28]. This copy number was strikingly smaller than that of *Saccharomyces cerevisiae* (~ 560) but similar to other filamentous fungi with compact architecture (Table 2), as well as most bacteria [29]. In *P. fructicola*, 44 tRNA genes were identified by tRNAScan-SE, similar to the total in *Pneumocystis jirovecii* (71tRNAs) and other *Pneumocystis* spp.(45 to 47 tRNAs) (9) but much less than that in *S. cerevisiae* or *T. deformans* (Table 2), or other eukaryotes (170–570 copies) [30].

**Table 2:** *Peltaster fructicola* nuclear genome statistics and comparison to other fungal species with highly compact genome

Species	*Peltaster fructicola*	*Taphrina deformans*	*Pneumocystis jirovecii*	*Saccharomyces cerevisiae*
Chromosomes (count)	5	NA^a^	NA^a^	16
Genome size (Mb)	18.99	13.3	8.1	12.07
GC content (%)	51.95	49.5	29.1	38.3
Protein coding genes (count)	8072	5735	3898	6002
Exons per gene (count)	2.32	2.1	3.7	1.13
Introns per gene (count)	1.36	NA^a^	4.7^b^	0.06
tRNA genes (count)	44	169	71	275
rDNA copies (count)	9	1	5	~ 560^b^
Intergenic distance (median, bp)	463	NA^a^	326	350
Intergenic regions (%)	31	36	29	26
Intron distance (median, bp)	50	NA^a^	45	111
Telomere repeat unit	TAGGG	TTAGGG	TTAGGG	T(G)_2–3_(TG)_1–6_
Repeat content (%)	0.34	1.5	9.8	5.13
Data source	Current study	From reference [8]	From reference [8]	Analysis in this study. NCBI R64 genome version was used

^a^NA, not applicable or not available from the website

^b^These data are obtained from reference [9]

### Reduced length of non-coding DNA

The median intron size of *P. fructicola* was 50 bp, only slightly more than that of *Pneumocystis jirovecii* (45 bp) and *Pseudocercospora fijiensis* (45 bp), but much less than the median of *S. cerevisiae* (111 bp) [31] and Dothideomycetes species in general (median = 57 bp, significantly different from *P. fructicola*) (Fig. 2). Intron size distribution in *P. fructicola* compared with others showed that the length of introns tended to be the shortest (Fig. S2). The longest intron size of *P. fructicola* was only 1053 bp, much short than others (1356 bp to 42,135 bp). Intron number of *P. fructicola* was significant higher than that of *S. cerevisiae* (Table 2), but the intron sizes in *P. fructicola* were strikingly smaller compared to *S. cerevisiae*. Intron size has been correlated to TE number [32], and as expected, *P. fructicola* also had a correlation between small intron size and fewer TEs.

Intergenic regions in *P. fructicola* occupied only 31% of the genome (Table 2), which was similar to that of *S. cerevisiae* (26%), *Pneumocystis jirovecii* (29%), and *Taphrina deformans* (36%), but smaller than for other Dothideomycetes species (range from 36% in *B. compniacensis* to70% in *Pseudocercospora fijiensis*). Moreover, 92.2% of the *P. fructicola* genome was covered by primary transcripts across all five stages. Genome-wide coverage of transcribed regions of the *P. fructicola* genome was significantly higher than for many non-compact fungal species, such as *Colletotrichum fructicola* (52.4%), *Passalora fulva* (60.4%), *Zymoseptoria tritici* (70.6%), *Alternaria brassicicola* (82%) and *Ustilago maydis* (84.0%), and even exceeded transcribed coverage for the human genome (~ 75%) [2]. Another SBFS fungus, *Ramichloridium luteum*, which shared some common features with *P. fructicola*, also had a high transcribed coverage (87.3%).

### Substantial reduction of the size of gene families but retention of all primary metabolic pathways

Based on a maximum likelihood tree constructed from concatenated alignment of 1957 single-copy orthologs conserved across 18 selected species, *P. fructicola* was clustered with other members from Capnodiales (Fig. 2a). We compared the *P. fructicola* genome with that of three other Capnodiales species with various lifestyles including the biotrophic plant pathogen *Passalora fulva*, hemibiotrophic plant pathogen *Z. tritici*, and saprotrophic fungus *B. compniacensis*. In an orthoMCL comparison between the four species, potential clusters of orthologous genes for comparative analyses were determined. A total of 139 genes (59 clusters) were unique to *P. fructicola* (orphans), and 5479 one-to-one orthologous genes (5337 clusters) of *P. fructicola* were identified. About 63% of the orphans were hypothetical protein-coding or had no homology to sequences in GenBank. Five orphans containing lipase_GDSL_2 domain (PF13472), a family of presumed lipases, were probably associated with colonization of the epicuticular wax layer on plant surfaces. *P. fructicola* had reduced cluster size; that is, it and *B. compniacensis* had the minimum number of multi-gene clusters (2 genes at least), compared with *Z. tritici*, *Passalora fulva* or *Saccharomyces cerevisiae* (Fig. S4). Some genes involved in transmembrane transport and hydrolase glycosyl chain activity were multi-gene clusters in *Z. tritici*, but there was only one copy of each cluster in *P. fructicola* (Table S4). Reduction in the size of gene families may be a key contributor to *P. fructicola*’s exceptionally diminutive genome among Dothideomycetes fungi. Although reduction in the number of gene families often occurred in *P. fructicola* [12], key metabolic pathways were completely retained, including genes involved in carbohydrate metabolism, amino acid metabolism, nucleotide metabolism, lipid metabolism and cofactor metabolism (Table S5, Table S6, Table S7, Table S8 and Table S9).

## Discussion

Sooty blotch and flyspeck (SBFS) fungi occupy an exclusively surface-dwelling niche. An ecologically distinctive group of plant pathogens, they have smaller genomes than their plant-penetrating parasite relatives [12–14]. The SBFS fungus *Peltaster fructicol*a was found to possess the smallest known genome size in Dothideomycetes [15], which is one of the largest groups of fungi with a high level of ecological diversity and life styles [15]. Expect, *P. fructicola* has higher gene density than other characterized Dothideomycetes species, only except for *B. compniacensis.* Comparing to fungi out of Dothideomycetes, *P. fructicola* also had similar gene density with highly compact fungal species *Pneumocystis jirovecii* and *Taphrina deformans* [8, 9]. Furthermore, by analysis of genome architecture, we confirmed that *P. fructicola* is not only the smallest but also the most compact fungus in Dothideomycetes, and highlighted the mechanisms associated with formation of its compact genome structure.

A remarkable feature of *P. fructicola* was a reduction in the number of repeats, a similar mechanism to that reported for the Ascomycota fungus *Pneumocystis jirovecii* [9], the basidiomycete yeast *Mixia osmundae* [33], the early-diverging fungus *Encephalitozoon cuniculi* [34], the algae *Ostreococcus tauri* and *Cyanidioschyzon merolae* [3, 35], the bladderwort plant (*Utricularia gibba*) [36], the pufferfish (*Tetraodon nigroviridis*) [37], and the Antarctic midge *Belgica antarctica* [38], whose small genomes are also characterized by compactness. An approximately 0.3% repeat content of the assembled *P. fructicola* genome was lower than that of *B. compniacensis* (0.8%) which was formerly the smallest fraction reported in Dothideomycetes [15]. Now, repeat content of *P. fructicola* was the smallest proportion reported not only in Dothideomycetes but also in the above eukaryotic compact species. Transposable elements (TEs) are enigmatic genetic units that play important roles in the evolution of eukaryotic and prokaryotic genomes [39]. Fungal genomes have varied TEs contents (from 0.7% of *Botrytis cinerea* to 70% of *Blumeria graminis* f. sp. *hordeï*) [39]. The 0.05% TE composition of *P. fructicola* shows substantial reduction. In particular, the number of retroelements was significantly fewer than that of the highly compact fungus *S. cerevisiae.* Avian malaria parasites *Plasmodium falciparum*, *P. knowlesi* and *P. relictum* are the only eukaryotes having no full-length TE detected in their genomes [40]. No full-length TE was detected in the *P. fructicola* genome, and all LTRs were fragments that entailed loss of all retroelementinternal sequences. *P. fructicola* may be the first fungus reported that contains no complete TEs. LTRs do not always lead to genome size expansion over evolutionary time, because there are also processes for rapid removal of DNA, such as from flowering plant genomes by accumulated deletions caused by illegitimate recombination [41–43]. Such recombination may also play an important role in genome shrinkage of fungal species*.*

Streamlining theory predicts that oligotrophic bacteria [30, 44] should have smaller genome sizes, with few rDNA copies, than non-oligotrophic bacteria. We found that the oligotrophic fungus *P. fructicola* followed this trend, containing just nine rDNA copies. It was generally assumed that rDNA redundancy allows the cell to maintain a functional ribosome in diverse environments and that a higher rDNA copy number allows for an increased rate of rRNA synthesis, resulting in a higher level of ribosome production and more rapid growth [45]. The reduction to nine rDNA copies in *P. fructicola* may reflect its genome stability as a result of adaptation to an extreme environment [46]. Few rDNA copies together with a minimal number of tRNA genes, suggest slow transcription and translation processes in *P. fructicola*, but growth efficiency could be high [29]. Interestingly, small and compact genomes of *Taphrina deformans*, *Pneumocystis jirovecii*, *Ostreococcus tauri* and *Cyanidioschyzon merolae* were achieved by this feature, with 1–5 rDNA copies, resulting in a strong positive relationship between rDNA copy number and genome size [25] or compact architecture. In the present study, the chromosome-scale assembly confirmed that TAGGG was the telomere repeat sequence in all *P. fructicola* chromosomal ends. In most filamentous fungi, the telomeres are composed of many copies of the sequence TTAGGG [47], and the *P. fructicola* telomere sequence, TAGGG, had never been found previously in fungal species. Most telomeric repeats range from 6 to 26 nucleotides [47], but the telomere repeat sequence of *P. fructicola* is composed of only 5 nucleotides, the shortest known telomere repeat unit among fungi.

Intron size has been positively correlated to TE number [32, 38], suggesting that small intron size of *P. fructicola* may be a result of fewer TEs. The intron size of *P. fructicola* was much less than that of a variety of genome sequenced Dothideomycetes species and the highly compact genome yeast *S. cerevisiae*. When compared to species outside the fungal kingdom, mean length of introns was much less than for the smallest free-living eukaryote, *Ostreococcus tauri* (103 bp) [3]. We suggest that reduction of intron size of the oligotrophic fungus *P. fructicola* might save a large amount of energy and resources used for intron processing. Moreover, intergenic regions in *P. fructicola* were the shortest among Dothideomycetes species, and the occupied proportion of the genome was close to that of *S. cerevisiae* and the compact yeast-like fungus *P. jirovecii* [9]. Shortening of intergenic regions and reducing intron size were clearly two major mechanisms behind the intense degree of genome compaction of *P. fructicola*. By transcribed coverage analysis of *P. fructicola*, *Colletotrichum fructicola*, *Passalora fulva*, *Zymoseptoria tritici*, *Alternaria brassicicola*, and *Ustilago maydis*, we suggest that its small introns and high relative contents of exon information may lead to a relatively high level of transcriptional coverage compared to these none compact fungal species examined. In the *S. cerevisiae* genome, loss of intron number was a major compaction mechanism. Therefore, we suggest that reduction of intron length rather than intron number in *P. fructicola*, *Taphrina deformans* and *Pneumocystis jirovecii* genome is the compaction mechanism. This is highly unusual in filamentous fungi.

Range of chromosome numbers in Ascomycota was 4 in *Fusarium graminearum* to 21 in *Zymoseptoria tritici* [21, 48]. Five chromosomes of the finished *P. fructicola* genome significantly reduced compared to 21 chromosomes in its related species *Z. tritici*. Compared to other species whose genomes are compact, a reduced number of chromosomes is a unique feature of *P. fructicola*, for example, 11 chromosomes in *Encephalitozoon cuniculi*, 16 in *S. cerevisiae*, and 20 in *O. tauri* and *C. merolae* [3, 34, 35]. Gene function of the five *P. fructicola* chromosomes was almost entirely non-redundant and only five collinearity blocks were detected. Cluster analysis of gene families revealed that *P. fructicola* had reduction in the size of gene families compared to non-compact genomes. Although genes involved in plant cell wall degradation, secreted peptidases and effectors were drastically reduced because the fungus does not interact with host immune defenses [12], all other major metabolic pathways were basically retained. It was different from the genome compact species *Pneumocystis jirovecii*, with substantial reductions of many metabolic pathways [9]. So we suggest that high gene density of *P. fructicola* appeared to constrain genome architecture, rather than gene content. Nevertheless, *P. fructicola* is an interesting anomaly among sequenced filamentous fungi in having a strikingly compact genome.

## Conclusion

In this study, we report the nuclear-genome sequence of *Peltaster fructicola* in a chromosome-scale assembly. Analysis of genome architecture and gene content revealed that *P. fructicola* genome possesses the smallest and the most compact genome in Dothideomycetes, and further yielded new insights into mechanisms for compaction in filamentous fungi. The intense degree of genome compaction in *P. fructicola* appeared to be the result of several processes. One major factor is the very low repeat content. Shortening of intergenic regions, small introns and very high coverage of transcribed regions are additional important factors. A reduced number of chromosomes and diminutive size of gene families also played roles in the intense genome compaction. Compaction mechanisms of filamentous fungus such as *P. fructicola*, *Taphrina deformans* and *Pneumocystis jirovecii* are distinct from those of yeasts such as *Saccharomyces cerevisiae*. Shortening of intergenic regions and reduction of intron size were clearly two major factors in the exceptional degree of genome compaction of *P. fructicola*. We considered that *P. fructicola* genome was highly compact by means of mechanisms that are distinct from those of *S. cerevisiae.* Interestingly, most of these genomic features of *P. fructicola* are shared by the extremophilic filamentous saprophyte *B. compniacensis* and another SBFS fungus, *Ramichloridium luteum*. Even some animals and plants that live in extreme environments exhibit genome reduction, and several kingdoms may share similarities in mechanisms of genome compact in such challenging habitats. Characterizing the exceptionally tiny genome of *P. fructicola* substantially broadens our understanding of the various compaction mechanisms in the fungal kingdom.

## Methods

### Biological sample

*Peltaster fructicola* strain LNHT1506 was obtained from a SBFS colony on the surface of a crabapple (*Malus* × *micromalus* Makino) fruit collected in Suizhong County, Liaoning Province, China. The cultures were purified by single spore isolation, maintained on potato dextrose agar (PDA) at 25 °C and stored as glycerol stock (15%) at -80 °C in the Fungal Laboratory of Northwest A&F University, Yangling, Shaanxi Province, China.

### DNA library preparation and Nanopore sequencing

DNA was extracted using the Qiagen® Genomic DNA Kit following manufacturer guidelines (Cat#13323, Qiagen). Quality and quantity of total DNA were evaluated using a NanoDrop™ One UV-Vis spectrophotometer (Thermo Fisher Scientific, USA) and Qubit® 3.0 Fluorometer (Invitrogen, USA), respectively. The Blue Pippin system (Sage Science, USA) was used to retrieve large fragments by gel cutting. DNA repair was performed using a purchased DNA repair mix (NEBNext FFPE DNA Repair Mix, NEB M6630). End repair and dA-tailing used NEBNext End repair/dA-tailing Module (E7546, NEB). Ligation was then performed by Ligation Sequencing Kit 1D (SQK-LSK108, Oxford). MinION sequencing was performed as per manufacturer’s guidelines using R9.4.1 flow cells (FLO-MIN106, ONT and controlled using Oxford Nanopore Technologies MinKNOW software. Flow cells were then transferred to Nanopore GridION × 5 (Oxford Nanopore Technologies, UK) for nanopore single molecular sequencing.

### De novo genome assembly

Canu v. 1.5 was used to assemble the Nanopore reads data set with default parameter [49]. One telomere was missing in a unitig by Canu assembly. To obtain a more complete assembly (telomere to telomere) the Nanopore data was analyzed along with Illumina data [12], and SPAdes v. 3.9.0 [50] was run with “-m 100 --nanopore nanopore.reads.fa --trusted-contigs canu.assembly.unitigs.fa -1Illumina.reads1.fastq -2 Illumina.reads1.fastq”. We then obtained a chromosome-scale assembly with all 10 telomeres filled in at the ends of the five unitigs. According to unitig size from largest to smallest, we defined chromosome names as pf_chr1 to pf_chr5. After the assembly step, we polished each set of unitigs with Pilon (v. 1.22) [51] using ~ 256× of Illumina 2 × 100 bp paired-end reads, and then used software Nanopolish v. 0.10.1 (https://github.com/jts/nanopolish) to do ‘second polish’ with nanopore fast5 files. To achieve a high-quality assembly (more complete and without gaps) assembly, we conducted a ‘third polish’ stage by using Pilon.

### Gene and repeat annotation

Gene annotation was accomplished using the BRAKER annotation pipeline to map expressed sequence tag evidence and ab initio gene predictions to the draft genome [52]. Filtered RNA-sequencing reads from a reference genome [12] and those sequenced in this study (GSE121872) were mapped to the genome with TopHat2 and putative transcripts were assembled with Cufflinks [53, 54]. The putative transcripts were used in BRAKER v.2.1.0 as expressed sequence tag evidence. The genome completeness of assembly was assessed using BUSCO v. 1.2 [55]. In all cases, we ran BUSCO in the protein mode, using the Fungi reference database with ‘-l fungi -m OGS’ parameter. Repeat sequences were identified by RepeatMasker v. 4.0.5 (http://www.repeatmasker.org) and RepeatModeler v. 1.0.7 [56] pipeline with RepBase library (version: 20170127). The syntenic information was detected by MCScanX [57] and drawn by Circos as circular plots [58]. Transcribed coverage was computed by all transcribed exons divided by genome size. When multiple isoforms were found, the shortest length of isoform was chosen for further analysis. The length of intergenic and intron was extracted and calculated by the in house PERL scripts.

### Protein family analysis

We identified ortholog pairs among selected fungal genomes by using OrthoMCL v. 2.0.9 (Table S10). To construct a genome-based phylogenic tree, MAFFT v. 7 was used to align single-copy ortholog pairs (http://mafft.cbrc.jp/alignment/server), conserved sites were extracted by using Gblocks v. 0.91b with the default parameters [59] and RAxML was used to construct maximum likelihood tree [60]. OrthoFinder was used to identify single or multi-copy genes from selected species [61].

### Transcriptome analysis

We designed five treatments including mycelia on potato dextrose broth (PD) for 5 d, mycelia on PD for 15 d, mycelia on PD with PEG 6000 for 15 d, mycelia on potato dextrose agar (PDA) for 15 d, and mycelia from artificial inoculations onto apple fruit in the field (15 d). Sequencing data from the latter two trials were published in our previous study [12].

For each sample, 10 μg of total RNA were used for RNA-seq library construction. Before being used for directional RNA-seq library construction, oligo (dT)-conjugated magnetic beads (Invitrogen) was used to purify and concentrate Polyadenylated mRNAs. Purified mRNAs were then iron fragmented at 95^o^ C and followed by end repair and 5’adaptor ligation. Next, reverse transcription was performed by using RT primer harboring 3′ adaptor sequence and randomized hexamer. After cDNAs were purified and amplified, PCR products were purified corresponding to 200–500 bps, quantified and stored at -80^o^ C until used for sequencing. The libraries were paired-end sequenced with a read length of 125 bp using Illumina HiSeq™ 2500 sequencing platform. After filtration, clean reads were mapped to the reference genome assembled in this study using TopHat v. 2.0.9 with the parameter “-g 1” [62]. TopHat was used to conduct mapping our sequenced species including *Peltaster fructicola*, *Ramichloridium luteum* [13] and *Colletotrichum fructicola* [63]. For other species in this study, we used Hisat2 v. 2.1.0 to map reads to their genomes with the parameter “--dta-cufflinks --sra-acc <SRA accession number>” [64]. SRA accession number was list in Table S11. A different gene expression profile was created using software Cufflinks v. 2.2.1 [53].

## Supplementary information

**Additional file 1: Table S1.** Genes in subtelomeric regions. ‘L’ means the left end of chromosomes. ‘R’ means the right end of chromosomes. **Table S2.** The annotations of collinear genes. **Table S3.** Repeat content in *Peltaster fructicola*. **Table S4.** Cluster containing unique gene in *Peltaster fructicola* but multi-genes in *Zymoseptoria tritici*. **Table S5.** Genes involved in carbohydrate metabolism. **Table S6.** Genes involved in amino acid metabolism. **Table S7.** Genes involved in nucleotide metabolism. **Table S8.** Genes involved in lipid metabolism. **Table S9.** Genes involved in cofactor metabolism. **Table S10.** Strain number and download source of selected species for phylogenomics. **Table S11.** Sequence Read Archive (SRA) accession number lists.

**Additional file 2: Figure S1.** Protein length distribution in *Peltaster fructicola*. Average length was 500 aa.

**Additional file 3: Figure S2.** Intron size distribution comparison. Box plot comparing the natural logarithm of intron size for the selected species and an outgroup member. Each box represents the interquartile range and outliers that are more than or less than 1.5 times the interquartile range are represented as dots in the boxplots.

**Additional file 4: Figure S3.** GO annotation information of *Peltaster fructicola* genome*.*

**Additional file 5: Figure S4.** Analysis of gene numbers and copies of *Peltaster fructicola*, *Baudoinia compniacensis*, *Zymoseptoria tritici*, *Passalora fulva* and *Saccharomyces cerevisiae*. Gene numbers of single copy and multi-copies are shown on the color bars.

## Data Availability

The genome assembly and gene annotation are available in the NCBI Genbank with WGS accession numbers of CP051139-CP051143. Raw sequencing reads are available in the NCBI Sequenced Read Archive under the accession numbers SRR11481813. The transcriptome data sets have been deposited at the Gene Expression Omnibus at the NCBI database under accession number GSE121872. The accession numbers obtained from NCBI database and subsequently analysed in this study are included in the Additional file 1 (Table S11).

## Abbreviations

BLASTN: Basic Local Alignment Search Tool Nucleotide
BUSCO: Benchmarking Universal Single Copy Orthologs
FPKM: Fragments Per Kilobase of transcript per Million fragments mapped
GO: Gene Ontology
SBFS: Sooty Blotch and Fly Speck
