# Supplementary figures and images for: A chromosome-scale assembly of the smallest Dothideomycete genome reveals a unique genome compaction mechanism in filamentous fungi
Source: BMC Genomics. 2020 Apr 23;21:321. doi: 10.1186/s12864-020-6732-8 (PMC7181583; doi:10.1186/s12864-020-6732-8)

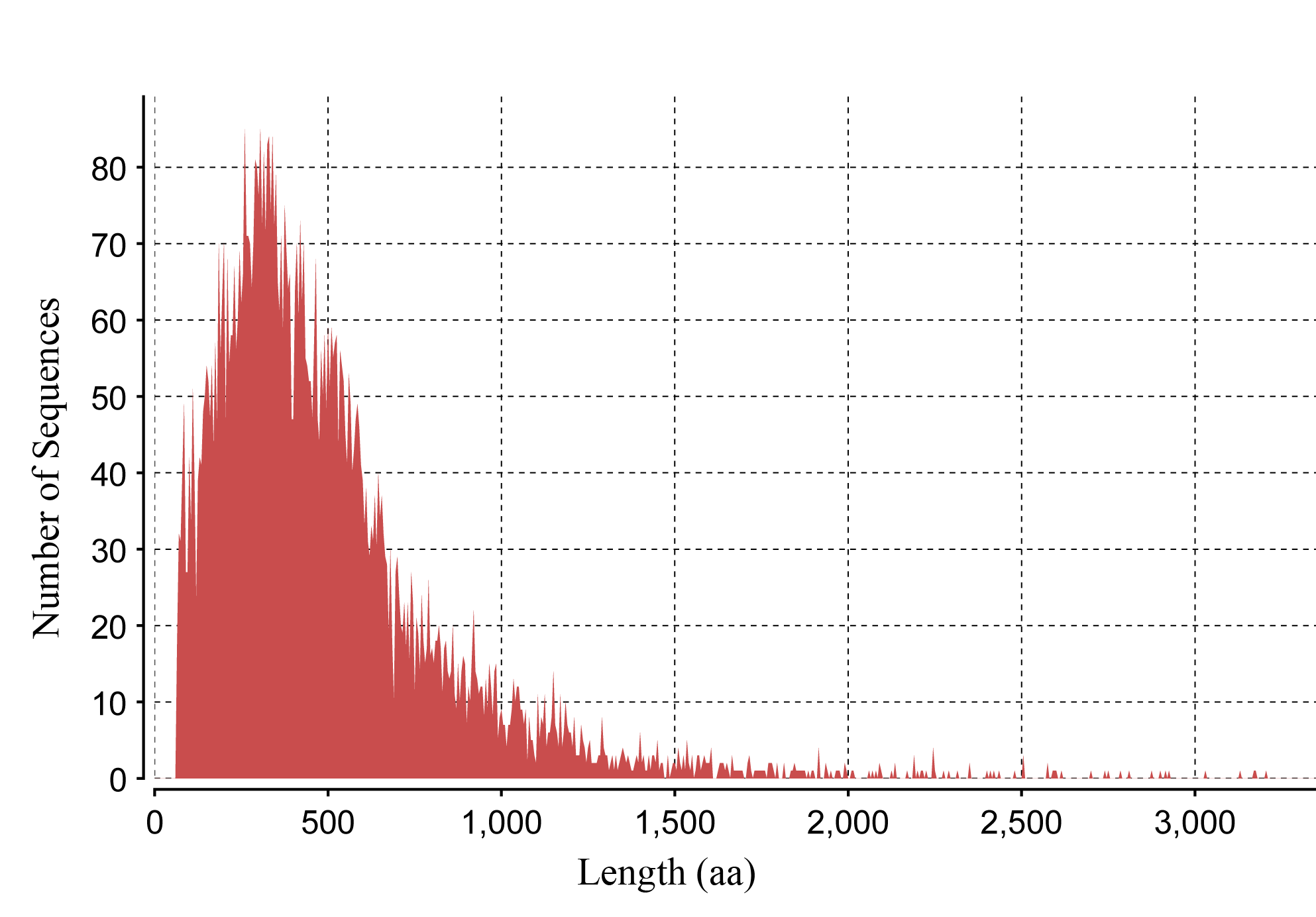

Supplement: Supplementary file 2 — Additional file 2: Figure S1. Protein length distribution in Peltaster fructicola. Average length was 500 aa. [file 12864_2020_6732_MOESM2_ESM.tif]

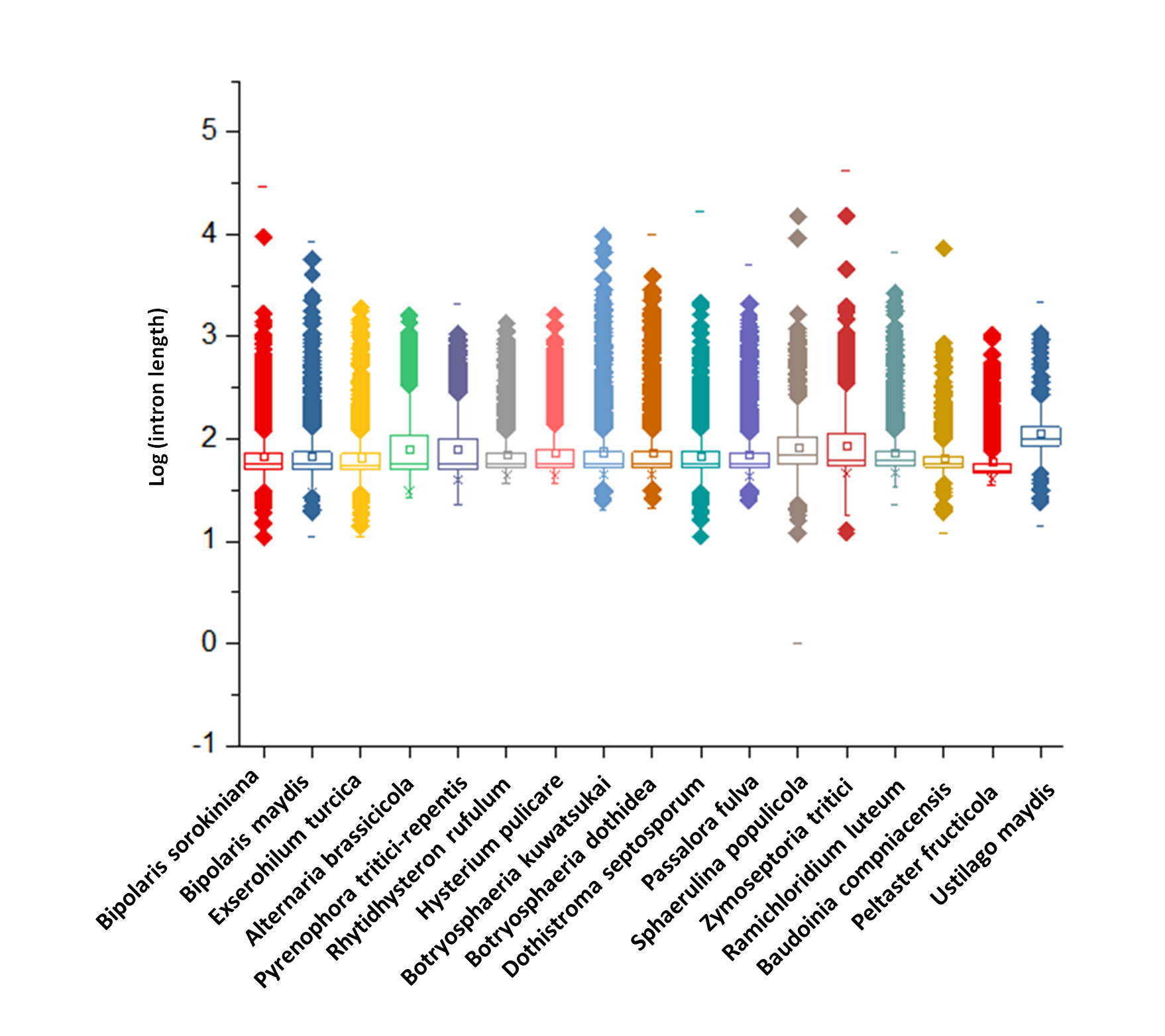

Supplement: Supplementary file 3 — Additional file 3: Figure S2. Intron size distribution comparison. Box plot comparing the natural logarithm of intron size for the selected species and an outgroup member. Each box represents the interquartile range and outliers that are more than or less than 1.5 times the interquartile range are represented as dots in the boxplots. [file 12864_2020_6732_MOESM3_ESM.tif]

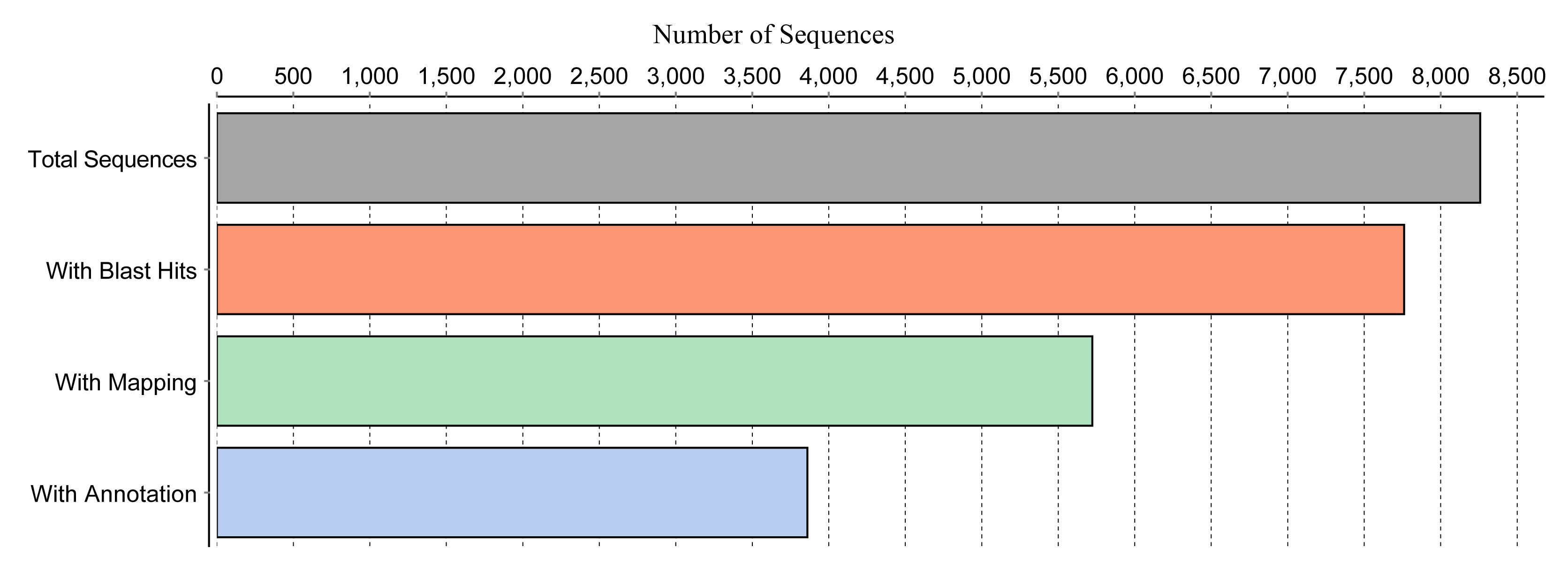

Supplement: Supplementary file 4 — Additional file 4: Figure S3. GO annotation information of Peltaster fructicola genome. [file 12864_2020_6732_MOESM4_ESM.tif]

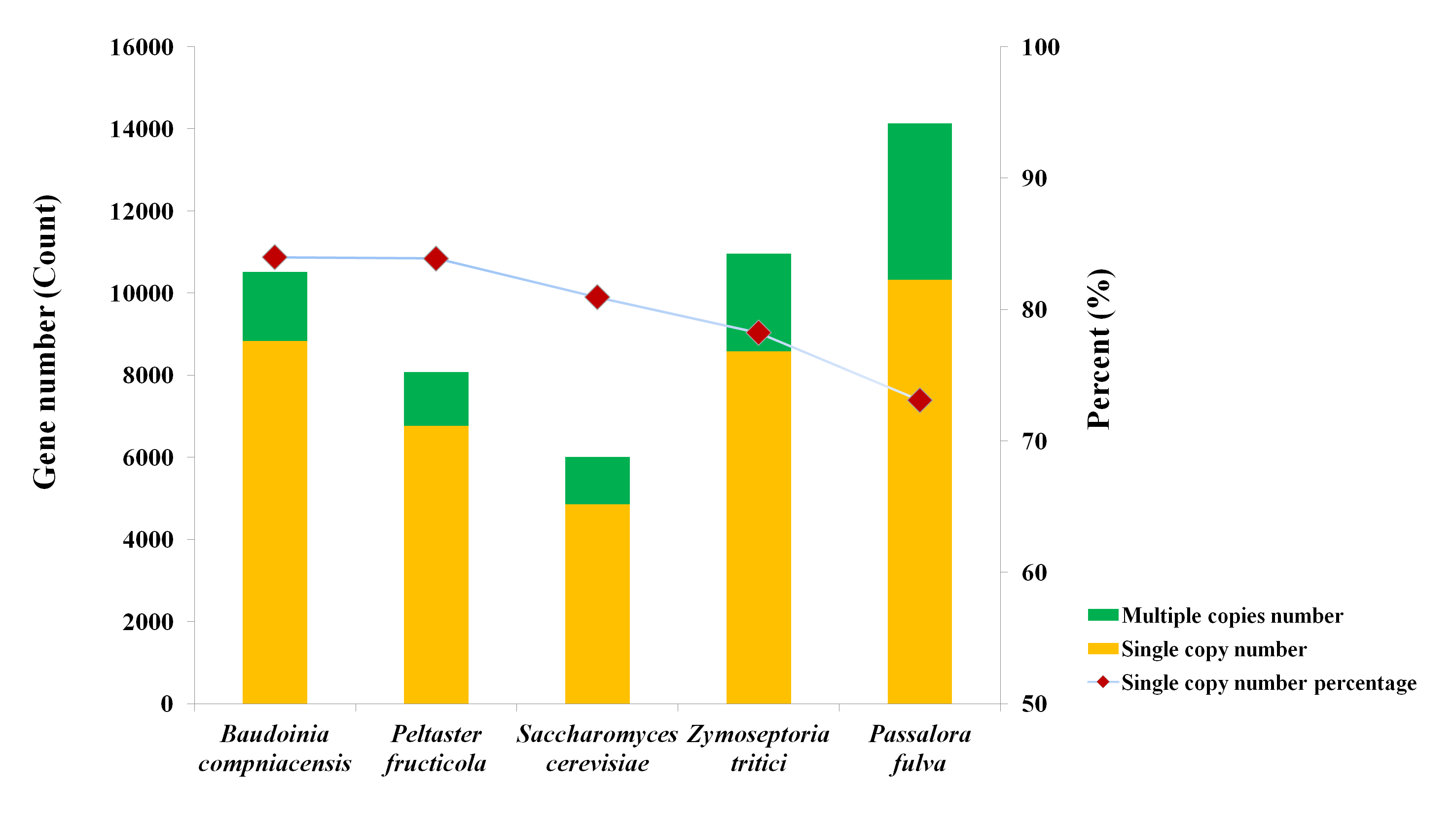

Supplement: Supplementary file 5 — Additional file 5: Figure S4. Analysis of gene numbers and copies of Peltaster fructicola, Baudoinia compniacensis, Zymoseptoria tritici, Passalora fulva and Saccharomyces cerevisiae. Gene numbers of single copy and multi-copies are shown on the color bars. [file 12864_2020_6732_MOESM5_ESM.tif]
